# Long Term Assessment of Predator Pressure on Artificial Nests of Common Pheasant ( *Phasianus colchicus* ) in Urban and Agricultural Areas of Lublin (Poland)

**DOI:** 10.1002/ece3.72336

**Published:** 2025-10-12

**Authors:** Piotr Czyżowski, Piotr Nawłatyna, Sławomir Beeger, Damian Zieliński

**Affiliations:** ^1^ Department of Animal Ethology and Wildlife Management, Faculty of Animal Sciences and Bioeconomy University of Life Sciences in Lublin Lublin Poland

**Keywords:** agricultural habitat, artificial nest, bird breeding, pheasants, predators pressure, urban habitat

## Abstract

In the study (2005–2023), the pressure of predators on breeding common pheasants ( *Phasianus colchicus* ) in urban (city of Lublin) and agricultural areas was compared using artificial nests. The average nest predation was higher in the urban than in agricultural areas. The main predator in the city was the common magpie ( *Pica pica* ), which preyed on nests mostly in ruderal areas and in the city park. The main predator in the agrocenoses was the red fox ( *Vulpes vulpes* ), which destroyed nests in the ecotone zones. A higher density of pheasants in urban areas, combined with a simultaneous higher pressure of predators than in agricultural areas, suggests that other environmental factors influence pheasant density more than the pressure of predators. In agricultural areas, the pressure of predators on artificial nests was shown to decrease along with the increase in the suitability of soils for agricultural production, which is associated with the decline in ecotone sites in intensively used agrocenoses.

## Graphical abstract

Higher pressure of predators on artificial nests is demonstrated in the urban area, compared to agricultural areas. The primary artificial nest predators are common magpies in urban areas and red foxes in agricultural areas.

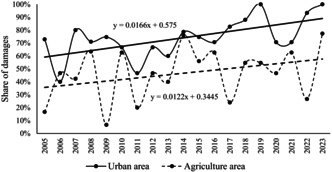

## 1 Introduction

Intensification of agriculture caused a decline in biodiversity of agricultural ecosystems, as a result of the establishment of large‐scale monocultures and elimination of mid‐field shelters, shrubs, ponds, etc., which in turn contributed to the destruction of natural sites and breeding grounds of wild animals (Stoate et al. 2001; Green et al. 2005; Henle et al. 2008; Lanz et al. 2018; Raven and Wagner 2021). These changes have made many wild species choose suburban areas for their refuge, which may provide better living conditions than adjacent agricultural areas (Fernandez‐Juricic and Jokimäki 2001; Zorenko and Leontyeva 2003; Cornelis and Hermy 2004; Kowarik 2011). Although most studies show that urbanization hurts the increase in biodiversity, research shows a decline in biodiversity mainly in the center of urban agglomerations and increasing biodiversity along the distance from the center toward the periphery of cities (Hermy and Cornelis 2000; McKinney 2002; Sorace and Gustin 2009; Concepción et al. 2015). With the increase in urbanization worldwide and the progressive fragmentation of the natural environment, the importance of urban green areas for biodiversity protection is growing. Urban and suburban areas can be treated as artificial ecosystems with specific habitat conditions, with urban parks, allotments, ruderal areas, etc., being the most critical component from an ecological point of view (Goddard et al. 2010; Kotze et al. 2011).

In research on the pressure of predators on bird breeding, artificial nests (dummy nests) that imitate natural broods are often used (Jokimaki and Huhta 2000; Zanette and Jenkins 2000; Thorington and Bowman 2003; Batary et al. 2004; Jones et al. 2005). Although long‐term studies of this subject are not so common. Many researchers (Haskell 1995; Weidinger 2001; Pärt and Wretenberg 2002; Zanette 2002) believe, however, that the use of artificial nests does not fully reflect actual predation patterns as the pressure of predators on artificial nests is different from the pressure on natural nests. Despite the criticisms of this method, artificial nests give results proportional to those provided by natural nests and are, therefore, a handy tool in comparative studies (Jokimaki and Huhta 2000; Thorington and Bowman 2003; Stephens et al. 2004). This has also been supported by more recent studies utilizing wildlife cameras, which confirmed that artificial nests can effectively reflect predation pressure across different landscapes (Krüger et al. 2018). In some aspects, research based on assessing predator pressure on dummy nests may have sound advantages over natural ones (see Major and Kendal 1996). For instance, it is possible to control the location of nests in different habitats and areas with varying urbanization or anthropic pressure. It is also possible to control the sample size, the number and size of eggs, and the nests' placement date.

The urban and suburban areas of the city of Lublin (SE Poland), that is, the city parks, allotments, ruderal areas, and meadows of the Bystrzyca river valley, provide habitats to numerous populations of wild species (Biaduń 1994; Czyżowski et al. 2011). These species have adapted to urban conditions characterized by intense human presence, many predators like, for example, the red fox ( *Vulpes vulpes* ), members of the Corvidae and Mustelidae families, seasonal mowing, noise, etc. (Holopainen et al. 2024). Due to the constant expansion of urban areas occupying animals' living space, many species choose areas located within the administrative borders of cities for their living places. One of the causes of the increase in their numbers is also the extensive mosaicism of the urban environment, expressed through forming a mix of city infrastructure and undeveloped, mostly rural landscapes (Kühn et al. 2004; Goddard et al. 2010). The common pheasant ( *Phasianus colchicus* ), whose density in the urban areas of Lublin is higher than in adjacent agrocenoses, is an example of such a species (Czyżowski and Karpiński 2010). Understanding the relationships between populations of wild animals inhabiting artificial ecosystems, such as urban areas, can help protect city biodiversity and provide crucial information on managing natural ecosystems. A unique role is played by research on bird populations due to their large numbers in cities, ease of observation, and high sensitivity to changes in the structure and composition of habitats (Savard et al. 2000; Sorace and Gustin 2009).

The work aimed to compare the pressure of predators on artificial pheasant nests in urban and adjacent agricultural areas. Higher density of pheasants in urban habitat, combined with a large number of predators in cities, might cause higher predator pressure than in agricultural areas. The growing number of animals in urban and suburban areas suggests that predator pressure will also increase there over the years.

## 2 Materials and Methods

The study was performed in 2005–2023. Artificial nests of pheasants were placed in urban and agricultural areas to imitate the natural ones of pheasant nesting, and systematic control of egg damage was carried out. Every year, 30 nests were set up (15 in urban areas and 15 in agricultural environments). Each of them had five non‐incubated small‐size and brown‐shelled chicken eggs. At the turn of April and May, the nests were placed in sites where pheasants were found by their calls. Dummy nests were placed in a small hollow without exceptional masking to resemble natural pheasant nests.

The research was conducted in Lublin's urban area and the Lublin region's agricultural communes in Poland. The urban area was located within the administrative boundaries of Lublin and covered allotment gardens, a city park, and ruderal areas. The agricultural area consisted of land in 16 municipalities of the Lubelskie Voivodeship, which differed in soil quality, terrain relief, and water conditions.

The nests in the urban area were placed in the same habitat every year (city park, allotment gardens, meadows of the Bystrzyca valley) but in different sites so as not to accustom potential predators to the location of the artificial nests. Artificial nests were placed yearly in different agricultural areas (communes). In 2 years (2007, 2008), two experimental plots were established in two different communes. Next, the average value obtained from the analyzed communes was accepted as the result from a given year. The sites where the nests were placed were randomly chosen, but the presence of shelter structures such as shrubs, balks, and others targeted the choice. The size of individual research areas on which the nests were located was approx. 500 ha, while the average distance between nests was approx. 400 m (based on the average distance between nests in 1979 Dumke and Pils study). All nests were marked with a number and placed so as not to catch the attention of predators. The nest number, location (marked on the topographic map), and inspection results were recorded. During the placing of the dummy nests, their distance (m) from the nearest buildings, watercourses, forest‐field borders, nearest Corvidae nests, as well as the number of common magpie ( *Pica pica* ) nests in their vicinity were also recorded. During the placement of the artificial nests in the urban and agricultural areas, the average density of pheasants (*n*/100 ha) was determined by counting cock roosting calls (Kamieniarz et al. 1992).

Artificial nests were inspected during the study four times: the first a week after starting the study and the next three at 1‐week intervals. During the inspection, the rate of egg depletion, traces made by the predator, and supplementary signs found around nests were recorded. These were the method of shell destruction (bendings of eggshell edges, marks on shell), traces and fresh scat left by the predator, characteristic odor left by some species, observed presence of potential predators in the study area, and other traces. Whole absent eggs were assigned to predator species that left their traces in the area surrounding the nest. The predation rate in individual controls was determined as a percentage of damaged eggs out of all placed eggs on a given habitat and estimated for all eggs laid (%) in the individual years in the compared areas and in individual inspections. The predators were ranked according to their decreasing effect on the artificial nests in the urban and agricultural areas. The average number of eggs in the nests and the nest's location (city park, meadow, mid‐field afforestation, etc.) were also presented.

In addition, the study attempted to assess the impact of selected environmental factors on the amount of predation on artificial nests in the subsequent years. To this end, information on meteorological data for the study area (average annual temperature, average monthly temperature, and precipitation of the spring period—March, April, May), as well as data on the agricultural areas (meteorological factors and quality of agricultural soils) were obtained from the Hydrological‐Meteorological Station in Radawiec (https://meteomodel.pl) and the Institute of Soil Science and Plant Cultivation in Puławy, respectively. Soil quality is based on assigning points to four elements of the natural environment: soil, climate, terrain, and water conditions. The sum of these points gives the indexation rate of agricultural production area; the higher the total sum of points for a given area, the higher the quality of agricultural production conditions (Table 1) (Witek et al. 1993).

**TABLE 1:** Index of agricultural production quality in individual municipalities (Witek et al. 1993).

Municipalities with artificial nests	Year of study	Index of agricultural production quality (points)
Strzyżewice	2005	87.1
Krzczonów	2006	94.7
Ludwin	2007	64.1
Wilkołaz	2007	91.7
Bełżyce	2008	86.3
Karczmiska	2008	71.7
Głusk	2009	90.7
Jabłonna	2010	88.2
Jastków	2011	100.8
Chodel	2012	70.2
Radecznica	2013	81.2
Konopnica	2014	103.1
Radzyń Podlaski	2015	69.2
Niemce	2016	85.5
Harasiuki	2017	46.0
Leśna Podlaska	2018	60.9
Janów Podlaski	2019	83.0
Piaski	2020	95.0
Bychawa	2021	92.9

### 2.1 Statistical Analysis

The significance of the difference in the percentage of damage between the urban and agricultural areas was tested using a two‐proportion *Z*‐test.

To assess the impact of multiple environmental factors on the extent of damage to artificial nests, a generalized linear mixed model (GLMM) analysis was conducted. All analyzed factors were included in the model: terrain type, landscape structure measures, and the presence of predator species as fixed effects, and nest location as a random effect. From among the many models, the best‐fitting one was selected, characterized by the largest number of statistically significant factors and the lowest Akaike information criterion (AIC) value.

The relationship between the average number of damaged eggs (*n*) in each municipality and the value of the agricultural production quality index in that municipality (points) was evaluated based on the calculation of Pearson's correlation coefficients.

The significance of differences between the average density of pheasants in the compared areas in the studied years was determined using the *t*‐test.

Conformity of distributions with the normal distribution was assessed using the Shapiro–Wilk test. Statistical analysis of the research results was done using Statistica 13.1.

## 3 Results

**FIGURE 1:**
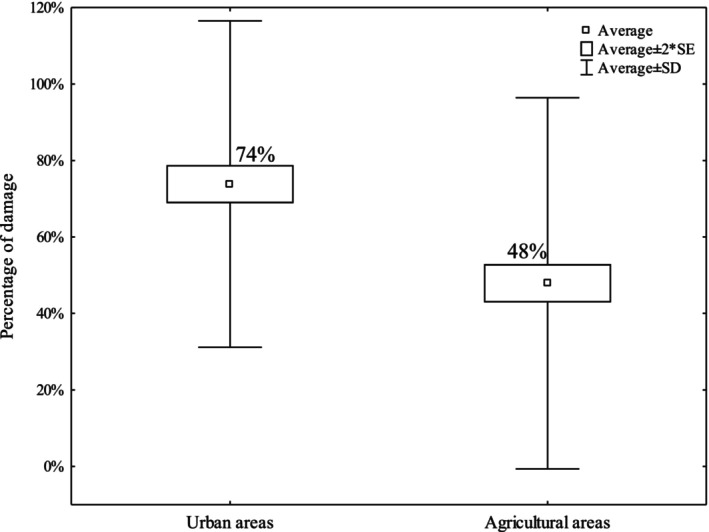
Predation rate (in percentages) in urban and agricultural areas throughout the study period (2005–2023).

**FIGURE 2:**
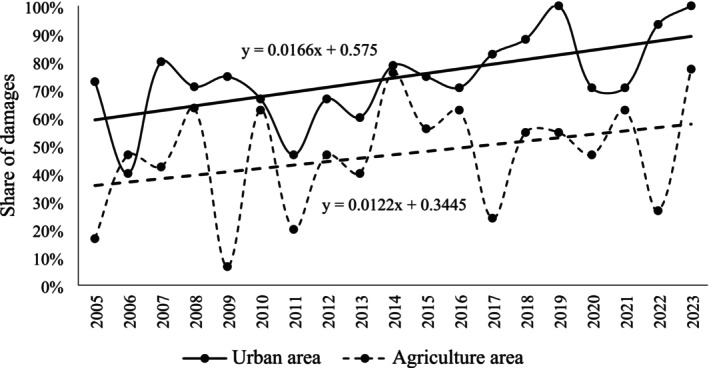
Comparison of the predation rates in the subsequent study years in the compared areas.

Throughout the study period, the average predation rate for all artificial nests was lower in the agricultural areas than in urban areas (Figure 1, *Z* = 13.55, *p* < 0.0001). This result is confirmed by the analysis of predation rates in the different years (Figure 2); in urban areas, the percentage of predated eggs exceeded 60% (except for 2006 and 2011), whereas in the agricultural areas, a more significant variability of the results was found, resulting primarily from the fact that the nests were established in different places in each study year. Over the years, placing eggs in different locations has allowed the phenomenon to be studied under different environmental conditions and has prevented predators from becoming accustomed to the feeding ground. Generally, in both studied areas, there was a tendency to increase the amount of damage throughout the research period, as evidenced by the plotted trend lines (Figure 2).

**FIGURE 3:**
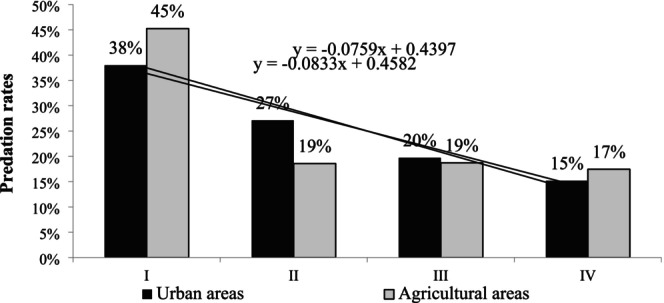
Nest predation rates in subsequent inspections in the compared areas.

**FIGURE 4:**
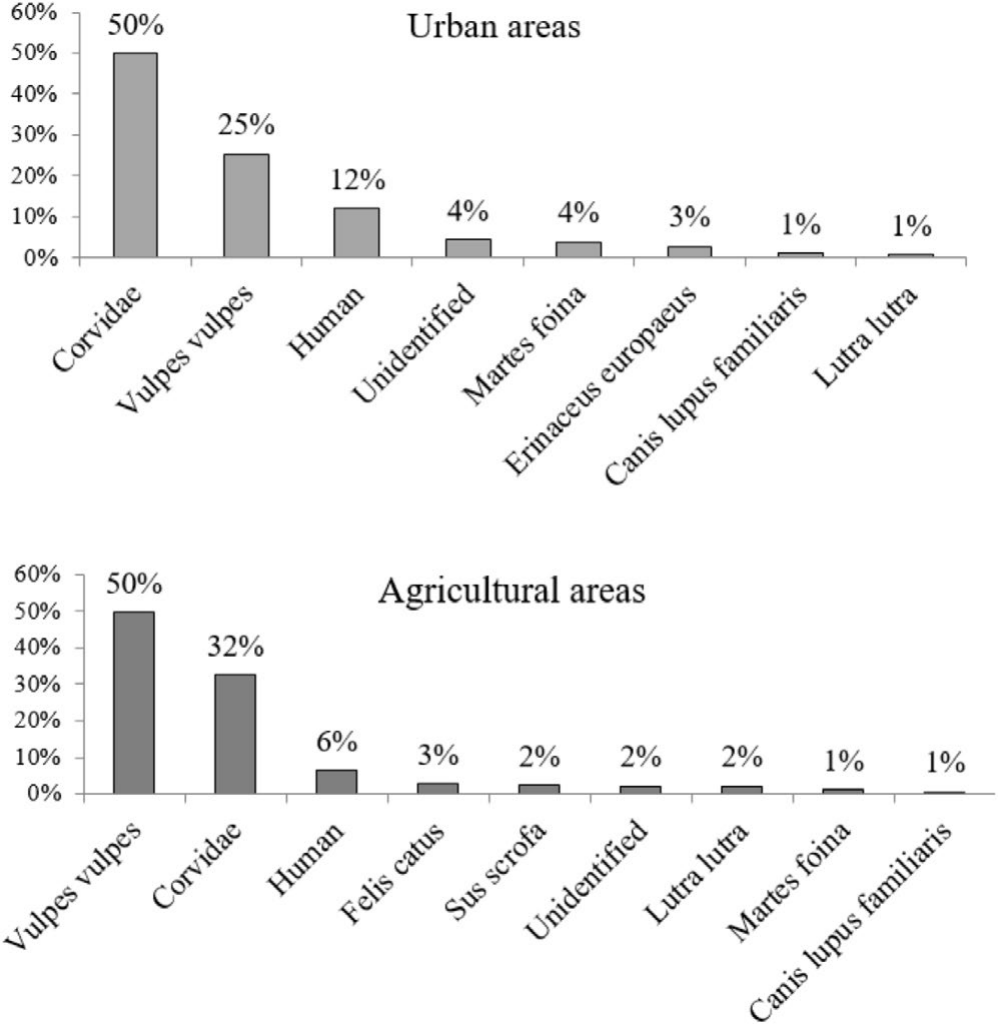
Percentage of eggs predated by different predators in the compared areas.

The analysis of predation rates in subsequent checks (Figure 3) revealed that most damage was done in both habitats in the first week of the observation. In the urban area, every inspection revealed a decline in the damage caused by predators. In turn, almost half of all damage in the agricultural areas was recorded in the first week and was followed by a significant reduction in predator pressure, which remained constant until the end of the research period. Corvidae, mainly the common magpie ( *Pica pica* ) and, to a lesser extent, the rook ( *Corvus frugilegus* ), were the dominant predators in the urban habitat (Figure 4). Damage done by these species was identified by the characteristic traces of broken eggshells and the observation of individuals and their nests around the damaged nest. The red fox ( *Vulpes vulpes* ) was responsible for one‐fourth of the urban predation, and the impact of this predator was recognized by the characteristic stench around the destroyed nest and the traces and droppings left behind. Man destroyed 12% of nests by grass mowing. Other predators were identified based on observations of their feeding near the nests (domestic dogs— *Canis lupus familiaris* , European hedgehog—*Erinaceus europaeus*) or characteristic feces (pine marten— *Martes martes* , European otter— *Lutra lutra* ).

The main nest predator in the agricultural areas was the red fox, which was responsible for half of all damage (Figure 4). Over 30% of the damage was done by corvids, mainly the magpie and, especially in the forest–field boundary, by the Eurasian jay ( *Garrulus glandarius* ). The rest of the damage was done by the same predators as in the urban area. An additional predator in the agrocenosis was the wild boar ( *Sus scrofa* ) and the domestic cat ( *Felis catus* ).

Nest predation rate showed significant differences in microhabitat location, as estimated by the statistical test for the two structure indices (Table 2). In the urban area, the biggest damage was observed in nests placed in city parks. Compared to other locations, it was a statistically significant rate. A more significant percentage variation in predated eggs was demonstrated in the urban area between the studied areas. The highest percentage of predation in agricultural areas was found among nests located in open areas (meadows, field bends), and this value was significantly higher than that of ecotones (mid‐field shelter, forest‐field boundary).

**TABLE 2:** Distribution of the percentage of predated eggs due to the location of the dummy nests.

	Urban areas	Agricultural areas
City park	81%	—
Meadow	69%	55%
Balk	—	57%
Ruderal area	68%	—
Mid‐field shelter	65%	45%
Allotments	61%	—
Field‐forest boundary	—	38%

**FIGURE 5:**
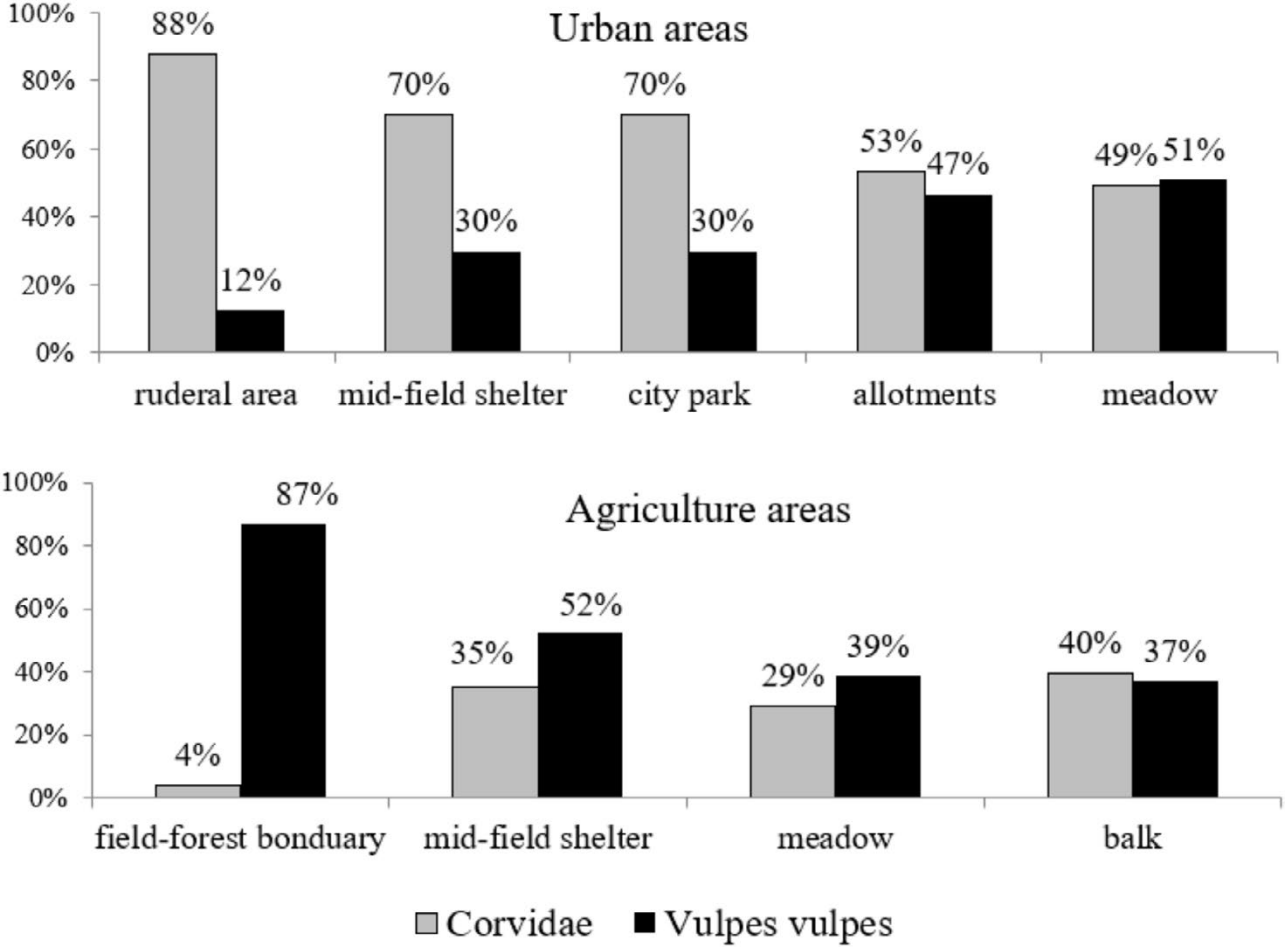
Percentages of eggs predated by the main predators (Corvidae and *Vulpes vulpes* ) due to the location of the nest in the compared areas.

The results showed differences in the contribution of the main predators, that is, Corvidae and the red fox *Vulpes vulpes* , to egg predation among locations (Figure 5). In urban areas, magpies are the main predator that destroy eggs. This species was responsible for most predation in the ruderal areas, mid‐field shelters, and the city park. On the other hand, the most minor damage caused by the red fox was noted in the nests placed in the city park, while the largest predation was found in the allotment areas and meadows adjacent to the Bystrzyca River.

The ANOVA results for the mixed model (Table 3) suggest that the distance of artificial nests from individual buildings significantly influenced the percentage of their destruction. This can be explained by the fact that the presence of humans and their infrastructure promotes greater activity of synanthropic predators, which tend to forage near human settlements. An even stronger influence on the percentage of destruction of artificial nests, according to the calculated model, was the distance of the artificial nest from natural magpie ( *Pica pica* ) nests. The proximity of this predator and the associated foraging activity increases the risk of destruction. This is also confirmed by the statistically significant effect of the number of magpie nests in the presented model.

**TABLE 3:** Results of the analysis of variance (ANOVA) for the mixed‐effects model with fixed and random effects, analyzing the impact of selected environmental components on the percentage of damage to artificial nests.

Variable	Efect	MS	*F*	*p*
Distance from single buildings (m)	Constant	0.7534	4.4620	0.0355*
Distance from villages or compact buildings (m)	Constant	0.5280	3.1268	0.0780
Distance from the field‐forest boundary (m)	Constant	0.3695	2.1883	0.1401
Distance from magpie nest (m)	Constant	1.4444	8.5542	0.0037*
Number of magpie nests	Constant	0.7141	4.2294	0.0406*
Nest location	Accompanying variable	0.1317	0.7799	0.5862
Urban/agricultural	Accompanying variable	0.3644	2.1580	0.1429
Year	Accompanying variable	0.3721	2.2034	0.0036*
Nests number	Random	0.1689		

* Significant for *p* ≤ 0.05.

The study did not show a statistically significant effect of distance from dense buildings or from the forest‐field border on the level of destruction, which may suggest that some landscape features have a more local effect rather than acting linearly with distance.

Significant differences between years may be related to seasonally changing environmental factors, such as food availability or fluctuations in the numbers of local predator populations. It is worth noting that this effect was statistically significant despite accounting for the random variability between nests, which indicates the considerable role of seasonal environmental variation.

In the presented mixed model, the differences in the level of destruction between urban and agricultural areas were not statistically significant. This result suggests that comparisons of predator pressure on breeding success in these areas should not be conducted without taking into account the local spatial structure of the landscape and the presence of specific predator species.

**FIGURE 6:**
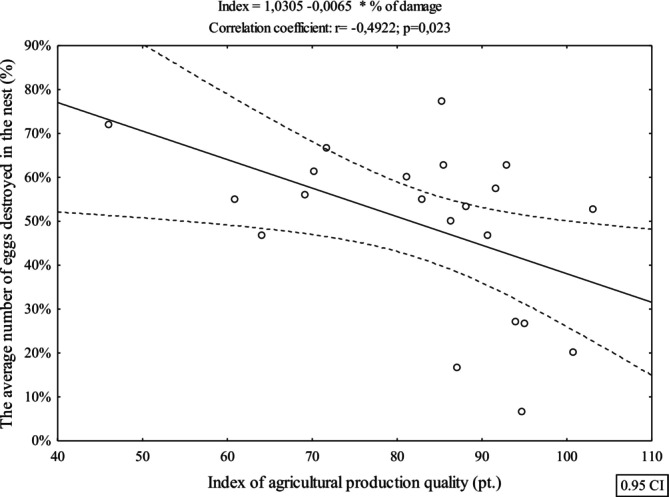
Relationship between the average number of preyed eggs in artificial nests and the value of the agricultural production quality index.

The location of the artificial nests in the agricultural areas in various communes allowed to determine the impact of agricultural conditions on predation rates. The research showed a significant impact of the agricultural production quality index (*r* = −0.50, *p* = 0.031), indicating that the increase in the value of the agricultural production quality index is accompanied by a significant decline in the average number of destroyed eggs in the artificial nests (Figure 6).

The annual inventory of pheasants showed a higher density in the urban areas than in the adjacent agricultural areas (Table 4).

**TABLE 4:** Average density of pheasants (*n*/100 ha) in 2005–2018 in the compared areas.

	Urban areas	Agricultural areas
Average density *n*/100 ha	5.6^A^	1.7^B^
SD	2.84	1.39
min	2.0	0.3
max	11.5	4.2

*Note:* Values marked with different uppercase letters (A,B) were statistically significant (*t* test = 4.612, df = 26, *p* = 0.0001).

## 4 Discussion

Nesting predation is considered to be the main factor reducing bird populations (Lima 2009), and this pressure is exceptionally high in urban areas where predation density increases (Gering and Blair 1999; Jokimaki and Huhta 2000; Sorace and Gustin 2009). According to Jokimaki et al. (2005), the pressure of predators in urban areas differs in individual countries. As shown by their research, the pressure of predators in urban areas in Finland was higher than in surrounding forests and assessed rural areas. There was no difference in predator pressure in Italy between urban, rural, and forest areas, while the pressure of predators in rural areas in Spain was higher than in cities. Our results indicate that even the decreasing distance of the artificial nest from individual buildings causes greater damage, probably due to increased activity of synanthropic predators. A similar effect was observed in the study by Fleming and Bateman (2018), where the presence of buildings increased the risk of nest predation. Jokimaki et al. (2005) showed that corvids were the main destroyers of nests in urban areas in Finland, while other predators, mainly dogs and cats, were indicated in Italy and Spain. Our study also showed a higher pressure of predators on pheasants in urban areas than in agricultural areas. Among the perpetrators of destruction in the city, corvids (mainly magpies) predominated, most often destroying nests in wooded areas (parks, sheds) and undeveloped ruderal areas in the city center.

Moreover, a negative and statistically significant relation was also found between the distance of artificial nests from the magpie nests in the urban areas. Therefore, the greater the distance between the artificial nest and the magpie nest was, the less damage the nest will suffer. This confirms that in urban areas, the main predator that destroys artificial nests is magpies. Due to the increase in the number of corvids in urban areas, mainly the magpie (Jerzak 1997), these birds are the main predators, destroying the breeding results of birds in cities (Gooch et al. 1991). Similarly, Bravo et al. (2023) demonstrated that the density of corvids significantly affects the increase in destruction of artificial nests of ground‐nesting birds. Our observations showed that the red fox was the second most effective destructor among the dummy nests in the urban area. This is consistent with the report presented by Draycott et al. (2008), in which the red fox and Corvidae were responsible for half of all pressure on breeding pheasants. In the agricultural areas, the most significant destruction was caused by foxes, which destroyed the nests in the ecotone sites (field‐forest boundary, tree plantations). As shown by other researchers (Santos and Telleria 1991; Šálek et al. 2010), such places are first searched by mammalian predators. The pressure of predators, especially foxes, is one of the leading causes of mortality among pheasants, although these birds are not their leading food (Millán et al. 2002; Tryjanowski et al. 2002; Baker et al. 2006). Another perpetrator of the destruction of nests in agricultural areas was the wild boar, which, according to Senserini and Santilli (2016), may be the main predator destroying pheasants' nests in areas of its abundant occurrence.

The steady growth in the size of the destruction of the artificial nests in the subsequent years demonstrated in this study was probably related to the increase in the number of magpies in the city of Lublin (unpublished data, Czyzowski P.), which confirms the overall increase in the density of this species in urban environments (Gregory and Marchant 1996; Suvorov et al. 2012). Studies carried out in SE Australia (Zanette 2002) and in three cities in Finland (Jokimaki and Huhta 2000) showed no annual variation in the magnitude of damage to artificial nests.

In our study, most nests were destroyed in the first week, which may be related to the insufficient vegetation development in early May, when predators can more easily find the nests. Studies conducted by Erdos et al. (2011) showed that damage to nests is lower if the nests are placed in high grass and more extensive plant cover. As shown in the research conducted by Mankin and Warner (1992), half of all artificial nests were damaged entirely within the first 2 days of observations.

Interestingly, the average number of destroyed eggs in the artificial nests was significantly reduced along with the increase in the value of the agricultural production quality index. The better the conditions for agriculture (better soils, water conditions, climate), the lower the pressure of predators on ground‐nesting birds, because the number of mid‐field trees and bushes decreases in heavily exploited agroforests. As shown in our research, the most significant pressure on agricultural areas was exerted by predators in open areas such as meadows or arable fields separated by balks, which dominate communes with better agricultural conditions. It is generally believed that arable fields are attractive to opportunistic predators such as the red fox and are regularly visited by them (Storch et al. 2005). As shown by the studies conducted by Tryjanowski et al. (2002), the growth of the fox population in intensively farmed areas has a more significant impact on the reduction of ground‐nesting populations than the decline in habitat diversity. In turn, as demonstrated in research carried out in Hungary (Purger et al. 2008), the dominant nest predators of these bird species (e.g., fox) prey mostly on small mammals; thus, the abundance of small mammals can influence the survival rates of ground‐nesting birds. Because rodents (Rodentia) from the genus of voles (Microtus) are the main component of the diet of many predators (Baker et al. 2006), an increase in their numbers in intensively used agrocenoses (Renwick and Lambin 2011) may decrease predator pressure on pheasant nestlings. It should also be considered that the survival of nests and the composition of nest predators are site‐specific and depend on the test method, as was also highlighted by recent studies (Krüger et al. 2018; Laux et al. 2022; Holopainen et al. 2024). Thus, it cannot simply be generalized (Praus and Weidinger 2010). Agricultural practices, particularly harvesting, were the leading cause of nest failure. Changes in agricultural practices would be a more effective way of increasing nesting success than predator control (Casas and Viñuela 2010).

### 4.1 Limitation of the Study

As mentioned in the introduction, the use of artificial nests to determine nest predators is a controversial topic. Many researchers believe that artificial nests do not fully replicate real pheasants' nests. Certainly, they differ from natural nests and stand out due to the absence of females. On the other hand, the use of artificial nests enables control of factors like the placement of nests that can also influence obtained results. These results may apply to other similar ground‐nesting bird species. While the study identified major predators (e.g., corvids, red foxes), it may not have captured all predator species involved in nest predation. Some predators might have been overlooked or not identified, particularly if they were elusive or caused less obvious damage. Additionally, the study did not explore the role of predator learning in nest destruction over time. Another consideration is that some of the urban areas used in the study might be, in fact, suburbs, if we compare building and human density there. Parameters that were not measured and might influence results were the number of people around dummy nests, as well as the visibility of artificial nests and surrounding plant cover. However, all nests were placed in as similar conditions as possible. These limitations suggest that further studies with more controlled conditions and broader geographic coverage are needed to draw more definitive conclusions about nest predation dynamics in urban and agricultural environments.

**FIGURE 7:**
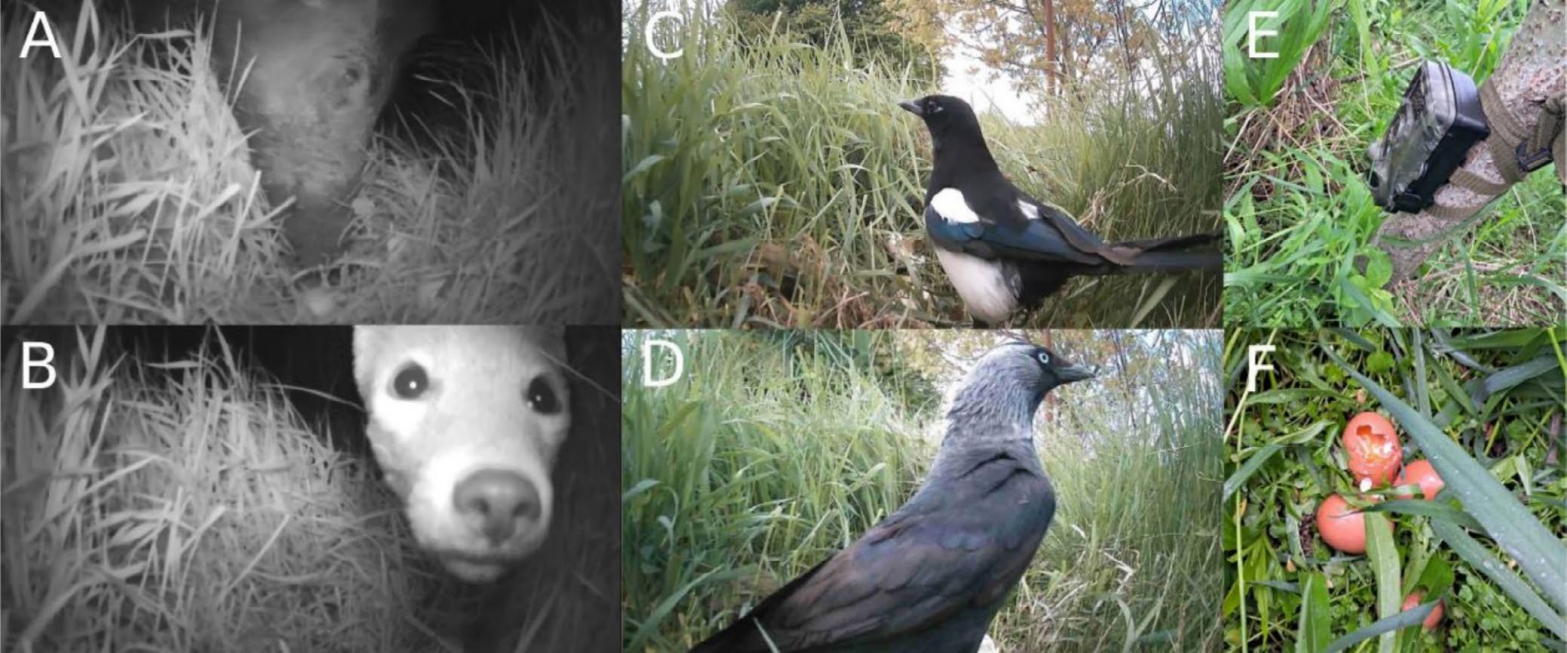
Predators photographed during damage to artificial pheasant nests. (A) wild boar ( *Sus scrofa* ); (B) red fox ( *Vulpes vulpes* ); (C) common magpie ( *Pica pica* ); (D) eurasian jackdaw ( *Corvus monedula* ); (E) one of the cameras used to photograph nests; (F) damaged eggs.

Our research to date has focused on analyzing traces and odors left on artificial pheasant nests during the breeding season, which has allowed us to tentatively identify potential species responsible for their damage. However, due to the lack of direct evidence of the sources of this damage, it was necessary to introduce more precise monitoring methods. During the current nesting season, we installed photo‐traps on the lined artificial nests, which made it possible to record the visits and behavior of animals visiting the nests in real time. Analysis of the recordings confirmed that the artificial nests were visited by a variety of species, including common magpies ( *Pica pica* ), red foxes ( *Vulpes vulpes* ), Eurasian jackdaws ( *Corvus monedula* ), and wild boars ( *Sus scrofa* ) (Figure 7). The presence of these species on the recordings provides direct evidence of their involvement in nest damage, confirming earlier assumptions about their role as the main culprits. In conclusion, the use of visual monitoring technology has significantly enriched our data, enabling us to unambiguously link specific species to damage to artificial nests. These results represent an important step toward a better understanding of interactions between wildlife and studies which are using artificial nests to link specific predator species to damage to wild pheasants' nests unambiguously.

## 5 Conclusions

Higher pressure of predators on artificial nests was demonstrated in the urban area, compared to agricultural areas. Higher density of pheasants in the urban area, compared to that in the neighboring agrocenoses and the simultaneous higher pressure of predators on the artificial nests in the urban area, suggests that other environmental factors than predator pressure exert a more significant influence on the density of pheasants. The main perpetrators of the destruction among pheasants are Corvidae—mainly *Pica pica* in the urban area and *Vulpes vulpes* in agricultural areas. In urban areas, predators mainly destroy nests in open areas, while their penetration in rural areas focuses on ecotone zones.

With the increase in the average monthly temperature, especially in spring months, the pressure of predators on birds' nests in urban areas increases. In agricultural areas, the pressure of predators on artificial nests was found to decrease along with the increase in the usefulness of soils for agricultural production, which may be associated with a decrease in the number of ecotone sites in intensively used agrocenoses.

## Author Contributions

**Piotr Czyżowski:** conceptualization (lead), data curation (equal), formal analysis (lead), investigation (equal), methodology (equal), supervision (lead), writing – original draft (equal). **Piotr Nawłatyna:** conceptualization (equal), data curation (supporting), investigation (equal), writing – original draft (equal), writing – review and editing (equal). **Sławomir Beeger:** conceptualization (equal), data curation (equal), methodology (equal), writing – original draft (equal). **Damian Zieliński:** conceptualization (equal), data curation (supporting), methodology (supporting), writing – review and editing (equal).

## Conflicts of Interest

The authors declare no conflicts of interest.

## Data Availability

The data that support the findings of this study are available on FigShare: https://doi.org/10.6084/m9.figshare.28417325.
